# Protocol for assessing phagocytosis activity in cultured primary murine microglia

**DOI:** 10.1016/j.xpro.2022.101881

**Published:** 2022-12-06

**Authors:** Elsie Layman, Jennifer Michelle Parrott, Hye Young Lee

**Affiliations:** 1 Department of Cellular and Integrative Physiology, University of Texas, Health Science Center at San Antonio, San Antonio, TX 78229, USA; 2 Department of Natural Science and Kinesiology, Northeast Lakeview College, Universal City, TX 78148, USA

**Keywords:** Cell Biology, Cell culture, Cell-based Assays, Microscopy, Neuroscience

## Abstract

In this protocol, we describe steps to assess inflammation-induced cell response in cultured primary murine microglia through the analysis of fluorescent bead phagocytosis. We detail primary murine mixed glial cell culture preparation followed by microglia-specific isolation. Further, we describe treatment with lipopolysaccharide (LPS) to induce phagocytosis of fluorescent beads, followed by quantitative analysis using fluorescent imaging and Fiji – ImageJ software.

For complete details on the use and execution of this protocol, please refer to Parrott et al.^1^

## Highlights

• A protocol for assessing phagocytosis activity of cultured primary murine microglia
• Dissection and culture of primary mixed glia cells from cortical tissue
• LPS-induced phagocytosis of fluorescent beads
• Characterization of microglia phagocytosis by fluorescent microscopy

## Abstract (editor highlights)

Publisher’s note: Undertaking any experimental protocol requires adherence to local institutional guidelines for laboratory safety and ethics.

## Graphical abstract

**Figure:**
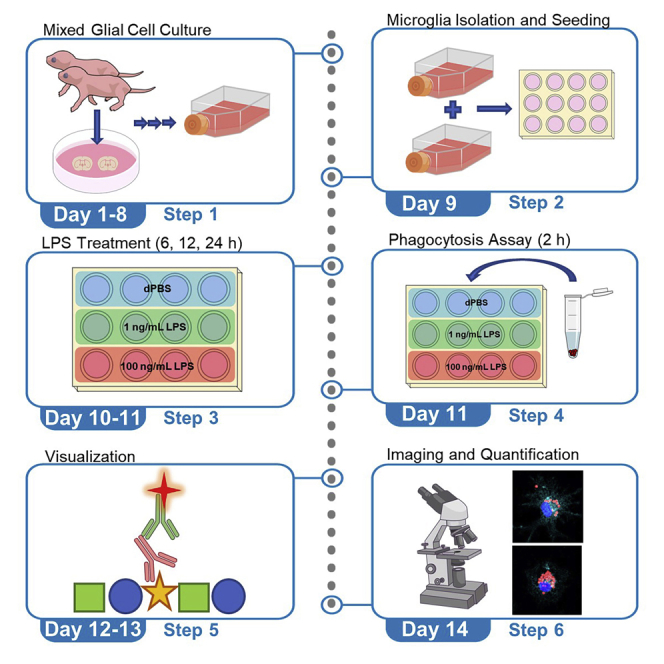

## Before you begin

This protocol was developed to understand and characterize brain cell differences between FVB.129P2-*Pde6b*^*+*^ *Tyr*^*c-ch*^/AntJ (wild-type) mice and FVB.129P2-*Pde6b*^+^ *Tyr*^*c-ch*^ *Fmr1*^*tm1Cgr*^/J (*Fmr1* KO) mice, a murine model to study fragile X syndrome (FXS). Specifically, the inflammatory response of primary microglia was assessed through the phagocytosis of fluorescent beads (Parrott et al.^1^). Breeding cages (one male mouse and two female mice) for each of the genotypes are maintained to generate mouse pups. To prepare primary mixed-glial cultures, male and female pups are sacrificed at a 1:1 sex ratio on postnatal day (PND) 2. Depending on the experimental purpose, please choose gender(s) accordingly. As described in Wolterink-Donselaar, Meerding and Fernandes (2009), PND 0 to 9 males can be distinguished from females by identifying the anogenital pigmentation found in the scrotum area.^2^ Cortical brain tissue from 4 pups of the same genotype, WT and *Fmr1* KO, are pooled to create a mixed-glial culture using previously established methods.^3^ This methodology of cortical dissection can be revised to obtain microglia from other brain regions of interest (such as the hippocampus) depending on the needs of the investigator.

This protocol has also been applied to pups at embryonic day (ED) 18/PND 0, PND 3, PND 7/8, and PND 10. It is important to note that tissue from PND 2 pup brains provided the most consistent and reliable results for the protocol described below. Typically, 2–6 pup brains (1:1 ratio of male:female pups) are used to create a culture per 75 cm^2^ flask, depending on availability of pups born in the same litter.

Prior to beginning this set of experiments, key resources (listed in the key resources table) should be available. Also, all the solutions described in the materials and equipment should be prepared.

### Institutional permissions

The use and care of animals in this study follow the guidelines of the University of Texas Health Science Center at San Antonio (UTHSCSA) Institutional Animal Care and Use Committee. Any health concerns or issues were handled by the veterinary staff maintained with UTHSCSA in the Department of Lab Animal Resources. Ethical guidelines and standards vary between different nations, states, and institutions. It is necessary to acquire permissions from relevant institutions to conduct the dissection outlined in this protocol.

## Key resources table

**Table:** 

REAGENT or RESOURCE	SOURCE	IDENTIFIER
**Antibodies**		

Anti-Iba1, rabbit (for immunocytochemistry), 1:500 dilution	FUJIFILM Waco Chemicals	Cat#019-19741; RRID: AB_839504
Rabbit IgG isotype control, 1:5000 dilution	Invitrogen	Cat#02-6102; RRID: AB_2532938
Cy™5 AffiniPure Donkey Anti-Rabbit IgG (H+L), 1:500 dilution	Jackson ImmunoResearch Laboratories, Inc	Cat#711-175-152; RRID: AB_2340607

**Chemicals, peptides, and recombinant proteins**		

DMEM (Dulbecco’s Modified Eagle’s Medium)	Corning, Inc.	Cat#10-013-CV
Gibco™ HBSS (Hanks’ Balanced Salt Solution, 1×)	Thermo Fisher Scientific	Cat#14170161
HEPES solution (N-(2-hydroxyethyl)piperazine-N’-(2-ethanesulfonic acid))	MilliporeSigma	Cat#H0887
Gibco™ Trypsin (2.5%), no phenol red	Thermo Fisher Scientific	Cat#15-090-046
Fetal Bovine Serum (FBS), Regular, USDA Approved Origin	Corning, Inc.	Cat#35-010-CV
Penicillin-Streptomycin Solution, 50×	Corning, Inc.	Cat#30-001-CI
Poly-L-Lysine (PLL), 100 mg	MilliporeSigma	Cat#P2636
ProLong™ Gold Antifade Mountant with DAPI	Invitrogen	Cat#P36931
Gibco™ Distilled Water	Thermo Fisher Scientific	Cat#15-230-147
Gibco™ DPBS, calcium, magnesium	Thermo Fisher Scientific	Cat#14-040-133
Goat Serum, New Zealand origin	Thermo Fisher Scientific	Cat#16210064
Hydrochloric Acid Solution, 1N (Certified), Fisher Chemical™	Thermo Fisher Scientific	Cat#SA48-500
Triton X-100	Thermo Fisher Scientific	Cat#A16046.AP
Deoxyribonuclease I (DNase) from bovine pancreas	MilliporeSigma	Cat#11284932001
Lipopolysaccharides (LPSs) from *Escherichia coli* O127:B8, purified by phenol extraction (Lot # 037M4067V)	MilliporeSigma	Cat#L3129
Latex beads, carboxylate-modified polystyrene, fluorescent red, aqueous suspension, 0.5 μm mean particle size	MilliporeSigma	Cat#L3280-1ML
Parafromaldehyde (PFA), 96%, extra pure, ACROS Organics™	ThermoFisher Scientific	Cat#416780010
10× PBS	Thermo Fisher Scientific	Cat#BP3994
Sodium chloride injection, USP 0.9%	Fresenius Kabi USA, LLC	Cat#918620
Sodium Hydroxide Solution (1N/Certified), Fisher Chemical™	Thermo Fisher Scientific	Cat#SS266-1
D(+)-Sucrose, 99.7%, for biochemistry, ACROS Organics™	Thermo Fisher Scientific	Cat#177140010

**Experimental models: Organisms/strains**		

Mouse: FVB.129P2-*Pde6b*^*+*^*Tyr*^*c-ch*^/AntJ, 1:1 sex ratio on postnatal day 2	Jackson Laboratory	004828; RRID: IMSR_JAX:004828
Mouse: FVB.129P2-*Pde6b*^+^*Tyr*^*c-ch*^*Fmr1*^*tm1Cgr*^/J, 1:1 sex ratio on postnatal day 2	Jackson Laboratory	004624; RRID: IMSR_JAX:004624

**Software and algorithms**		

GraphPad Prism	GraphPad	https://www.graphpad.com/scientific-software/prism/
Fiji – ImageJ	Fiji – ImageJ	https://imagej.net/software/fiji/
Zeiss ZEN software	Zeiss	https://www.zeiss.com/microscopy/int/products/microscope-software/zen.html

**Other**		

28 mm Diameter Syringe Filters, 0.2 μm Pore PES Membrane, Sterile, Individually Packaged, 50/Case	Corning, Inc.	Cat#431229
Falcon® 70 μm Cell Strainer, White, Sterile, Individually Packaged, 50/Case	Corning, Inc.	Cat#352350
50 mL PP Centrifuge Tubes, Conical Bottom with Plug Seal Cap	Corning, Inc.	Cat#430290
15 mL PP Centrifuge Tubes, Rack Packed with Plug Seal Cap	Corning, Inc.	Cat#430052
10 mL Stripette™ Serological Pipets, Polystyrene	Corning, Inc.	Cat#4100
Cole-Parmer Parafilm PM996 Wrap, 4″ Wide; 125 Ft/Roll	Cole-Parmer	Cat#0672040
Posi-Click 1.7 mL Microcentrifuge tubes	Thomas Scientific	Cat#1159M35
Petri Dish 100 × 15MM, 500/Case	Corning, Inc.	Cat#351029
Flasks 75 cm^2^ U-shape vent (T-75)	Corning, Inc.	Cat#430641U
Costar® 12-well Clear TC-treated Multiple Well Plates, Individually Wrapped, Sterile	Corning, Inc.	Cat#3513
Coverglass 18MM/100PK	Warner Instrument Corp	Cat#640384
Superfrost™ Plus Microscope Slides	ThermoFisher Scientific	Cat#12-550-15
1300 Series A2 Class II, Type A2 Biological Safety Cabinet	ThermoFisher Scientific	Cat#1323TS
Thermo Scientific™ Forma™ Series II Water-Jacketed CO2 Incubator, 184 L	ThermoFisher Scientific	Cat#3110
Vacuum Aspirator Bottle, 0.5 gal (1.9 liter)	Bel-Art Products	Cat#F19917-0001
Bottle/Tube Roller	ThermoFisher Scientific	Cat#88881003
TMS Inverted Microscope	Nikon	Cat#212373
Stereo Microscope SZM series	Guangzhou Hanker Electronics Technology	Cat#ba-008
CM-7S Plus Benchtop Centrifuge	Elmi	Cat#CM-7S
Nutating Mixer	ThermoFisher Scientific	Cat#05-450-213
Colibri 7	Zeiss	https://www.zeiss.com/microscopy/int/products/microscope-components/lightsources/colibri-7.html
Axio Observer with Apotome	Zeiss	https://www.zeiss.com/microscopy/int/products/light-microscopes/axio-observer-for-biology.html
LSM 710 Confocal Microscope	Zeiss	https://www.zeiss.com/microscopy/en/products/light-microscopes/confocal-microscopes.html

## Materials and equipment

**Table:** 50× PLL

Reagent	Final concentration	Amount
Poly-L-Lysine (PLL)	50×	50 mg
DPBS (dPBS), w/calcium, w/magnesium	–	10 mL
**Total**		**10 mL**

Filter using a 0.2 μm filter. Prepare 1 mL aliquots. Store at −20°C, stable for 6 months.

**Table:** 1× PLL

Reagent	Final concentration	Amount
50× Poly-L-Lysine (PLL)	1×	1 mL
DPBS (dPBS), w/calcium, w/magnesium	–	49 mL
**Total**		**50 mL**

Store at −20°C, stable until expiration date shown on label, thaw at 4°C before plating.

**Table:** 1× PBS

Reagent	Final concentration	Amount
10× PBS	1×	100 mL
Double-distilled H_2_O	–	900 mL
**Total**		**1 L**

Filter using a 0.2 μm filter. Store at 18°C–26°C, stable for 6 months.

***Note:*** 10× PBS solution contains 1.37 M NaCl, 0.027 M KCl and 0.119 M phosphates.

**Table:** 4% PFA

Reagent	Final concentration	Amount
Parafromaldehyde (PFA), 96%, extra pure, ACROS Organics™	4%	40 g
1× PBS	–	1,000 mL
**Total**		**1 L**

***Note:*** Warm 650 mL 1× PBS to 55°C and add 40 g PFA. Cover with aluminum foil to prevent PBS from evaporating and stir until solution becomes clear while maintaining the temperature at 55°C. Allow PFA solution to cool to 18°C–26°C, then adjust volume to 1 L with 1× PBS. Filter using a 0.2 μm filter and aliquot PFA into 50 mL conical tubes. When protected against light, filtered 4% PFA aliquots can be stored at −20°C for several years, 4°C for several weeks, or 18°C–26°C for 1–2 weeks.

**CRITICAL:** When dissolving the PFA into 1× PBS, make sure the temperature remains constant at 55°C. Deviations can prevent complete dissolution of PFA or can interfere with the chemical structure. Prepare PFA under a fume hood.

***Note:*** If the PFA fails to dissolve when warmed, add 1 N NaOH dropwise to the solution until the solution is clear. After filtering the solution, use a pH probe to adjust solution pH to 6.9 with filtered 1 N NaOH or filtered 1 N HCl.

**Table:** HBSS-H

Reagent	Final concentration	Amount
Hanks’ Balanced Salt Solution, 1× (HBSS)	–	19.8 mL
HEPES solution, 1 M	10 mM	0.2 mL
**Total**		**20 mL**

Make immediately before dissection, store on ice.

**Table:** Heat-inactivated FBS

Reagent	Final concentration	Amount
Fetal Bovine Serum (FBS)	–	500 mL
**Total**		**500 mL**

***Note:*** Thaw the bottle of fetal bovine serum (FBS) at 4°C for 8–18 h. Prepare a water bath with enough autoclaved DI water to completely immerse the FBS serum bottle but not the bottle cap. Heat the water bath to 56°C using a thermometer to ensure the bath maintains this temperature. Gently swirl the bottle to ensure the serum is homogenous and place the bottle in the water bath. Continue to gently swirl the serum every 5 min to ensure the solution remains homogenous. After heating the serum for 30 min, cool the serum bottle on ice. When the serum reaches 18°C–26°C, filter with a 0.2 μm filter and aliquot into 50 mL conical tubes. 50 mL aliquots of the filtered, heat-inactivated serum can then be stored at −20°C for several years.

**Table:** 10% FBS/DMEM/PS

Reagent	Final concentration	Amount
Dulbecco’s Modified Eagle’s Medium (DMEM) 1×	–	440 mL
Heat-inactivated Fetal Bovine Serum (FBS)	10%	50 mL
Penicillin-Streptomycin Solution, 50×	1×	10 mL
**Total**		**500 mL**

Store at 4°C, stable for 3 months.

**Table:** 50 mg/mL DNase

Reagent	Final concentration	Amount
Deoxyribonuclease I (DNase) from bovine pancreas	–	50 mg
Double-distilled H_2_O		1 mL
**Total**		1 mL

Filter using a 0.2 μm filter. Prepare 100 μL aliquots and store at −20°C, stable for 1 year. Thaw at 4°C before dissection.

**Table:** 1% FBS/DMEM/PS

Reagent	Final concentration	Amount
Dulbecco’s Modified Eagle’s Medium (DMEM) 1×	–	48.5 mL
Heat-inactivated Fetal Bovine Serum (FBS)	1%	0.5 mL
Penicillin-Streptomycin Solution, 50×	1×	1 mL
**Total**		**50 mL**

Store at 4°C, stable for 3 months.

**Table:** 1 mg/mL LPS

Reagent	Final concentration	Amount
Lipopolysaccharides (LPS) from *Escherichia coli* O127:B8, purified by phenol extraction	–	5 mg
Sterile Saline	–	5 mL
**Total**		**5 mL**

Store at 4°C for up to 1 month.

**CRITICAL:** Sterile saline and a sterile glass vial are necessary to prevent contamination when preparing LPS. It is also important to not filter the LPS stock as the polysaccharides can stick to the filter and change the concentration of the stock. A glass vial is also necessary for this reason as the polysaccharides stick to plastic impacting the final concentration of LPS.

**Table:** Blocking Solution

Reagent	Final concentration	Amount
1× PBS	–	18.99 mL
Goat serum	5%	1 mL
20% Triton X-100	0.01%	10 μL
**Total**		**20 mL**

Filter using a 0.2 μm filter. Store at 4°C up to 1 month.

## Step-by-step method details

### Dissection set-up

**Timing: 10 min**

1. In a 50 mL conical tube, prepare 20 mL of HBSS-H as described below under materials and equipment.
2. Add 10 mL of HBSS-H to a 15 mL conical tube. Add the remaining HBSS-H to a petri dish for tissue dissection. Keep both the conical tube and petri dish on ice.
3. Thaw 100 μL DNase and 500 μL 2.5% trypsin at 4°C.

***Note:*** This set-up is intended for the dissection of 2–4 pups for one culture. If preparing multiple genotypes at once, repeat this set-up for each genotype and label as necessary.

### Dissection and tissue collection

**Timing: 2 h**

***Note:*** Prior to beginning dissection, 10% FBS/DMEM/PS media should be prepared (see materials and equipment) and an aliquot of about 20 mL should be incubated at 37°C.

***Note:*** A video demonstrating cortical dissection similar to the method described below has been previously published.^4^ Please note this video example uses embryonic pups as opposed to PND 2 pups.

**Figure 1:**
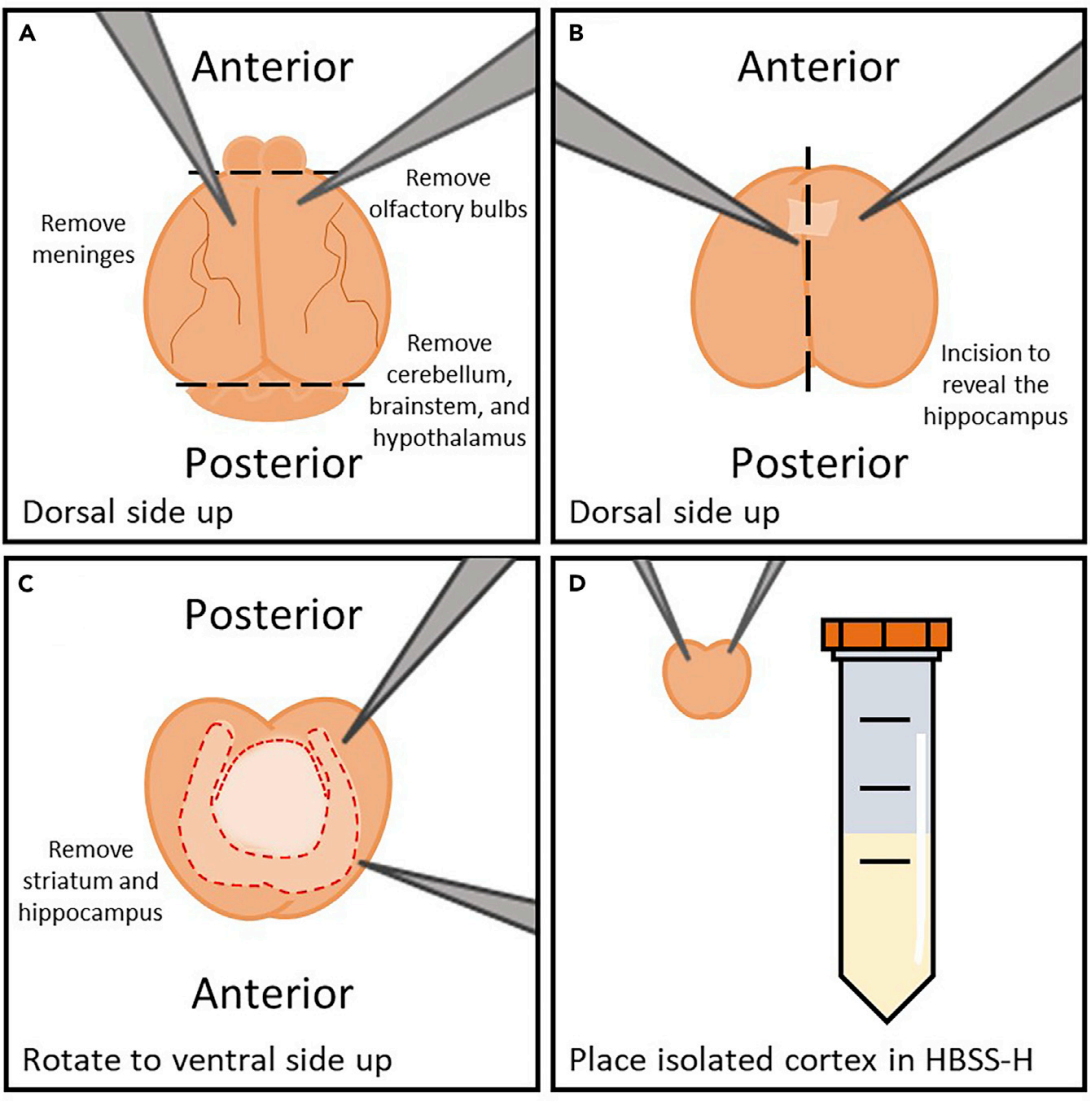
Illustration of brain dissection and collection of the cortex (A) After removing the murine brain from the skull, remove olfactory bulbs, meninges, cerebellum, brainstem, and hypothalamus. (B) Carefully cut into the midline of the brain, avoiding cutting all the way through, peeling the cortex away to reveal the hippocampus. (C) Flip the brain from the dorsal to ventral side, revealing the striatum. Remove the striatum. Cut along the corpus callosum and peel tissue to reveal hippocampus. Remove hippocampus to isolate cortex. (D) Ensuring all meninges are removed from cortex, collect cortex into prepared 15 mL conical tube containing HBSS-H.
7. Using a dissecting scope, dissect out the hippocampus and striatum to isolate the cortex (Figures 1B and 1C). Make sure to keep the tissue/HBSS-H cold.
  a. With the ventral side of the brain exposed and the anterior of the brain to the top, use forceps gently roll the cortical layer up to expose the hippocampus.
  b. From the corners created where the two cuts meet at midline, roll back the ventral part of the cortex away from midline to expose the hippocampus.
  c. Invert the brain (posterior of the brain to the top) and slide curved forceps under the hippocampus to find the lateral edge.
  d. Disconnect the hippocampus on both lateral sides from the cortex underneath. Also, separate the hippocampus from the tissue that is on the inferior edge.
  e. Identify the striatal tissue (inferior to the hippocampus) and use the curved forceps to remove the striatum as well. Be careful to only remove the superficial portion of the tissue as there is more cortex underneath the striatum.
8. Collect the cortical tissue pieces from the petri dish and place into the designated 15 mL conical tube containing 10 mL HBSS-H (Figure 1D).

***Note:*** (Optional) The collected tissues can be cut into small pieces with forceps or razor blades before placing into the conical to aid the trypsinization process.

4. Decapitate the pup to separate the head from the body.
  a. Spray the head of the pup with ethanol and grip back (dorsal side) of the body tightly to pull the arms outward.
  b. Flip the pup so that its stomach (ventral side) is facing up.
  c. Quickly decapitate the pup with sharp scissors.
5. Separate the brain from the skull to prepare for brain dissection.
  a. Gently remove the skin from the top of the skull (dorsal side) by pulling skin away from the eye sockets with forceps.
  b. Cut open the skull along the midline. Begin at the brainstem opening and continue to the anterior portion of the skull (near eye sockets).
  c. Peel the two sides of the skull back from the midline cut until the skull has been removed from the top of the brain tissue.
  d. Use a small scoop tool to remove the brain from the base (inferior portion) of the skull.
  e. Place the brain in the petri dish containing HBSS-H and keep on ice.
6. Remove the meninges, cerebellum/brainstem, olfactory bulbs, and hypothalamus with forceps (Figure 1A).

9. Repeat dissection and tissue collection (steps 1–5) for all pups, collecting all the cortical tissue together in the same conical tube of HBSS-H.
  a. Keep samples on ice during collection.
  b. For an additional genotype, collect tissues into a second separate conical with HBSS-H.

***Note:*** 2–4 pups are typically used for culturing in a 75 cm^2^ (T-75) flask. 1 pup dissection will take approximately 5–10 min. For 4 pups, the dissection may take up to 40 min.

***Note:*** During dissection, tail tissue can be collected from pups and can be used for genotyping to confirm assumed genotype.

***Note:*** If planning to dissect many pups (greater than 6), consider performing multiple dissections so that the tissue remains on ice for no longer than 60 min.

### Mixed glial cell culturing

**Timing: 1 h**

**CRITICAL:** Glial culturing should be conducted in a sterile environment, ideally a cell culture hood.

***Note:*** DNase should be prepared before beginning culture (see materials and equipment).

10. Aspirate (using a vacuum suction system) HBSS-H from the conical tube containing the collected cortical tissue.

***Note:*** Make sure tissue is settled at the bottom of the conical tube prior to beginning aspiration.

**Figure 2:**
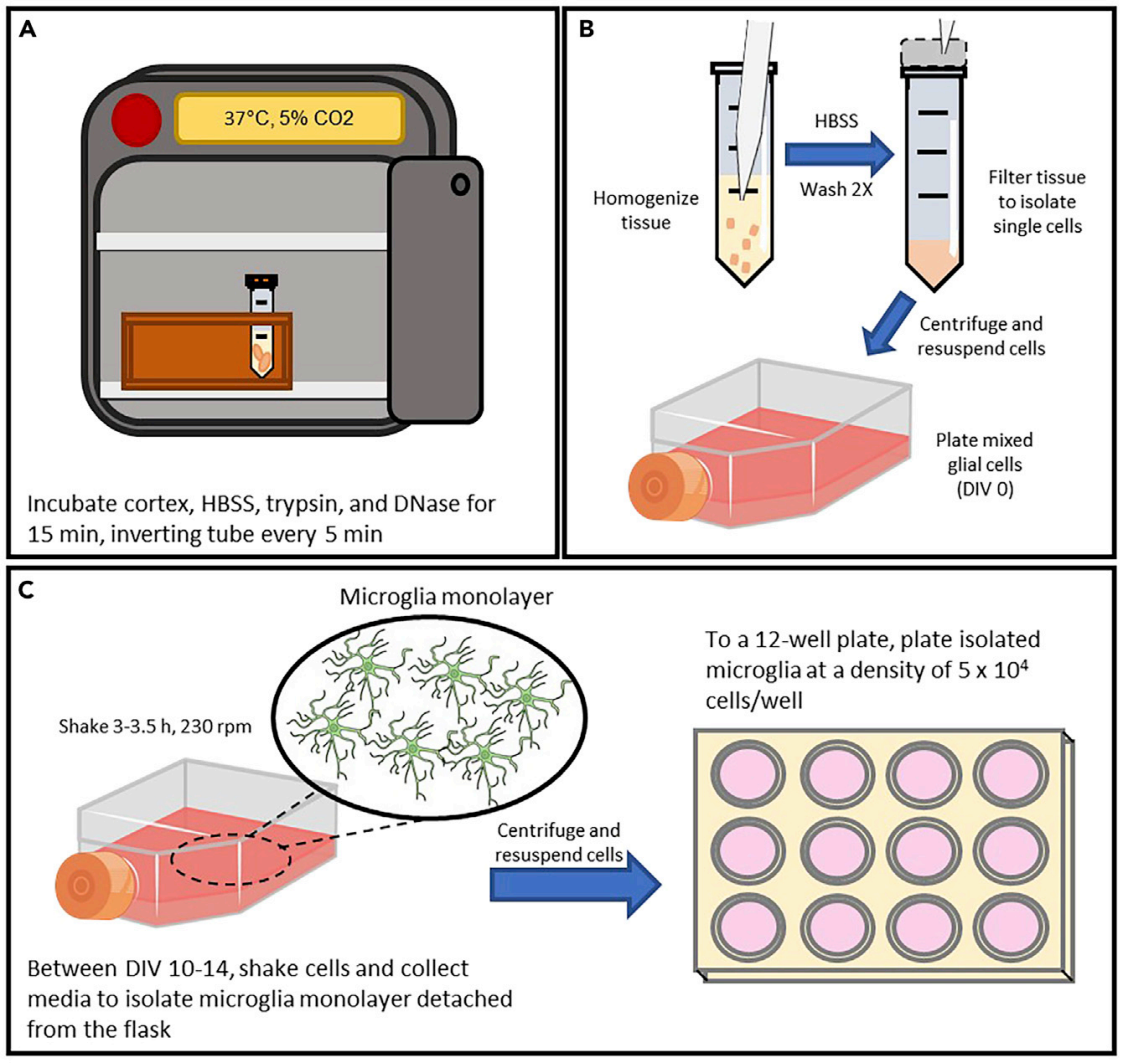
Primary mixed glial culture, shaking, and seeding microglia (A) After collecting the cortices from 2–6 pups, incubate cortices with trypsin, HBSS, and DNase at 37°C for 15 min. Every 5 min, invert tube. (B) After completing incubation, homogenize the tissue with P1000 and P200 pipettes, then filter the tissue to isolate single cells. These cells may then be plated with warmed media in a T-75 flask. (C) At DIV 10–14, cover the flask with aluminum foil and shake cells at 230 rpm for 3–3.5 h. Shaking of the flasks will detach microglia, which adhere in a loose monolayer on the flask. Collect the media containing microglia and plate cells to a 12-well plate.

11. Add 4 mL HBSS, 500 μL 2.5% trypsin, and 70 μL DNase to the conical tube with the cortical tissue and invert to mix well (Figure 2A).
  a. Incubate in 5% CO2 incubator at 37°C for 15 min, inverting every 5 min to mix the cortical tissue and the HBSS/Trypsin/DNase solution.

**CRITICAL:** Monitor incubation at this step closely. It is important to not over incubate the cortical tissue with trypsin.

12. After the final inversion, wait for the cortical tissue to settle to the bottom of the conical tube then aspirate the HBSS/Trypsin/DNase solution.
13. Wash the cortical tissue twice with 10 mL HBSS, inverting each time to wash completely.

***Note:*** Make sure tissue is settled at the bottom of the conical tube after inverting and prior to beginning each aspiration.

14. Add 2 mL HBSS and 30 μL DNase to the conical tube with the cortical tissue. With a P1000 pipette, homogenize the cortical tissue by slowly pipetting up and down 10 times (Figure 2B).
  a. Homogenize the cortical tissue again with a P200 pipette, slowly pipetting up and down 10 times.
15. To a 50 mL conical tube, add 2 mL of 10% FBS/DMEM/PS media (warmed to 37°C).
16. Place a 70 μm cell strainer on top of the 50 mL conical tube. Wet the strainer with 10% FBS/DMEM/PS media to minimize cell loss or cell death upon first contact with the strainer surface.
17. Transfer homogenized cortical tissue using a P200 pipette.

**CRITICAL:** A 70 μm cell strainer is required to isolate single cells from tissue.

18. Centrifuge isolated cells/media in the conical tube for 4 min 30 s at 300 rcf.
19. Aspirate media from the conical tube being careful to avoid the cell pellet.Resuspend isolated cells in 5 mL of 10% FBS/DMEM/PS media (warmed to 37°C).
20. To a T-75 flask, add 15 mL of 10% FBS/DMEM/PS media (warmed to 37°C).
21. Pipette resuspended isolated cells into the T-75 flask and media.

22. Place the T-75 flask with media/isolated cells into a 37°C cell culture incubator (sterile and maintained at 5% CO_2_).
  ***Note:*** Approximately 2.5–3.75 × 10^6^ dissociated single cells are expected per cortex dissected from one mouse pup (PND 1–4) when seeding cells.^5^
  a. Day of culture is day *in vitro* 0 (DIV 0).

### Mixed glial culture maintenance

**Timing: As long as appropriate for the experiment (typically DIV 14–21)**

**Figure 3:**
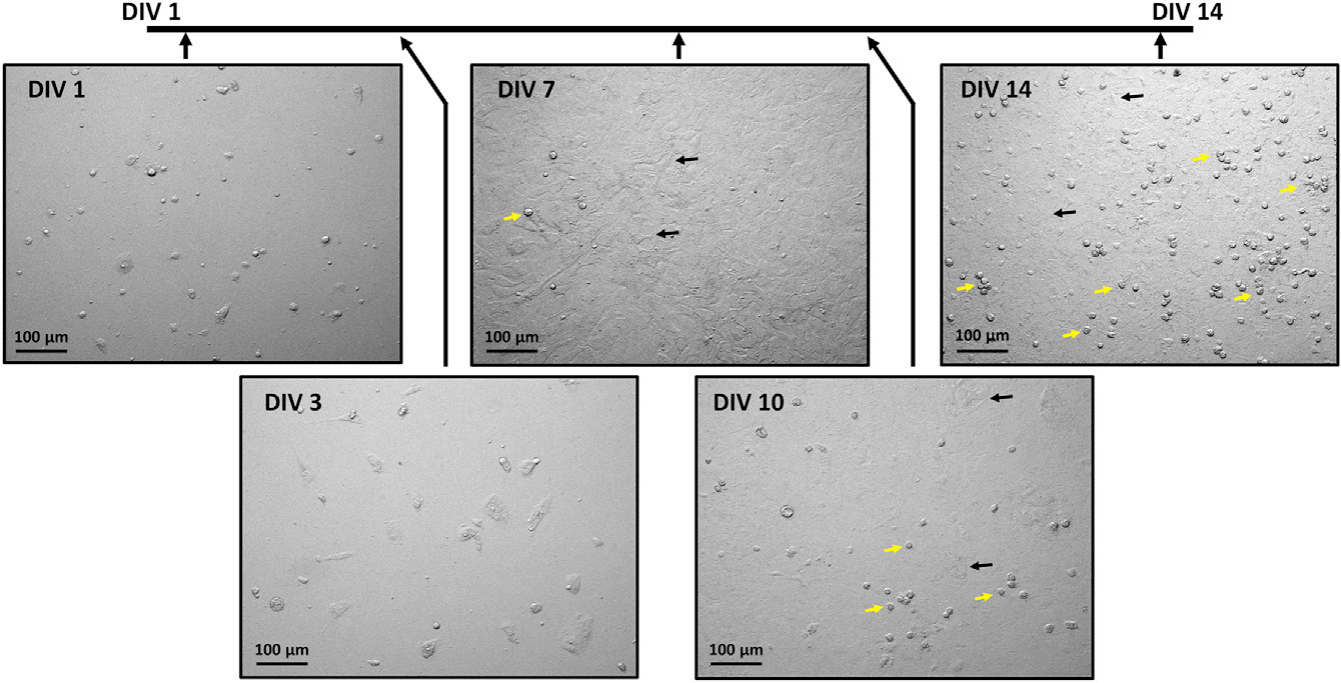
Timeline of mixed glial culture development (DIV 1–14) During DIV 1–7, a bottom layer consisting of astrocytes forms. Microglia begin forming clusters and individual cells above this astrocytic layer. At about DIV 10, microglia become dense and float above astrocytes (mainly). By DIV 14, microglia have become very dense. Yellow arrows indicate examples of microglia development. Black arrows indicate examples of astrocyte development. Scale bars for all panels represent 100 μm. Images taken at 10× magnification.

***Note:*** A visualization of the development of the mixed glial culture at different timepoints (DIV 1–14) can be found in Figure 3.

23. After at least 24 h (DIV 1), the media should be aspirated and replaced with 20 mL of new 10% FBS/DMEM/PS (warmed to 37°C) to remove any debris.
24. At DIV 4 or DIV 5, the media should be changed again to remove any additional debris.
  a. Additional media changes should be done each week after this point.

**CRITICAL:** Whenever replacing media, the new media should be incubated at 37°C for about an hour to avoid cold shock in the culture.

***Note:*** Microglia should have DIV 10–14 prior to collection for an experiment. Example of healthy cell culture development from DIV 1–14 are visualized in Figure 3. During growth period, check for culture attachment and health with a microscope. If microglia fail to grow by DIV 14 or appear unhealthy, please consult troubleshooting problem 1.

### Preparing plate for microglia plating

**Timing: 10 min hands-on, 2 h total**

25. Prepare a 12-well plate for the assay 30 min before beginning microglia shake-off.
  a. To each well, add one 18 mm glass coverslip.
  b. Add 600 μL 1× poly-l-lysine (PLL), described below under materials and equipment, to each well. Agitate the plate to make sure the cover glass is completely submerged.
26. Incubate the 1× PLL-coated plate for at least 2 h (at 37°C).
  a. Every hour, agitate the plate ensure cover glass is well coated.
27. Near the end of the microglia shake-off, aspirate the 1× PLL and wash the coated wells with cell culture grade water. Aspirate and repeat wash for a total of two washes with cell culture grade water.
  a. The plate is now ready to be plated with cell/media suspension.

***Note:*** Wait to remove the 1× PLL from the 12-well plate until cell/media suspension is ready to be added. If necessary, leave 1× PLL in 12-well plate and incubate at 37°C longer than 3 h. However, it is not ideal to leave plate incubating for much longer, so timing is important.

### Collection and seeding of microglia

**Timing: 1 h hands-on, 25 h total**

**Figure 4:**
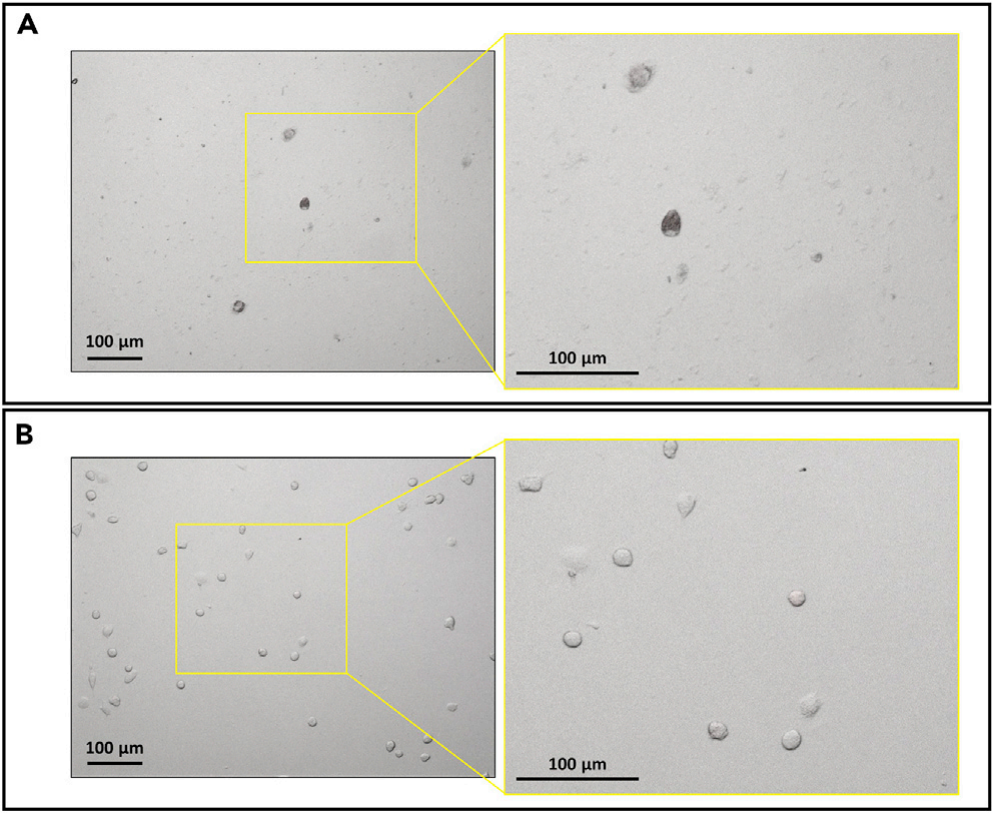
Examples of unhealthy and healthy isolated microglia after shaking Microglia were plated with a density of 5 × 10^5^ cells/well in a 12-well plate. Images were taken three days after plating. (A) Unhealthy primary cultured microglia: unhealthy cells have notable debris with a lower density of cells. Cells appear darker with a less defined amoeboid shape than healthy cells. (B) Healthy primary cultured microglia: healthy cells have minimal debris with a more defined amoeboid morphology. Scale bars for all panels represent 100 μm. Higher magnifications of the left images (yellow boxes) are shown on the right panels. Images taken at 10× magnification.
29. Shake the T-75 flasks at 230 rpm at 18°C–26°C for 3 h - 3 h 30 min. Check after 3 h shaking under the microscope and decide whether they will need longer shaking.
30. Once the shake-off is complete, immediately collect the media containing microglia from the flasks with a pipette.
  a. Shaken-off cells should be collected into a 50 mL conical tube.

***Note:*** Shaking-off microglia from the non-adherent floating cell layer is a commonly used method to isolate microglia from primary mixed glial cultures.^6^^,^^7^ This method yields high-purity microglia cultures, with an expected purity of > 90%.^8^

***Note:*** Between DIV 10–14, microglia are ready for collection. To prepare a full 12-well plate with an appropriate number of microglial cells, plan to seed two T-75 glial cultures. Examples of healthy, viable cells and unhealthy cells after plating can be found in Figure 4.

28. Cover the T-75 flasks containing mixed glia culture to be shaken in aluminum foil. Place the covered flasks on shaker and securely attach the flasks with tape (Figure 2C).

**CRITICAL:** After shake-off, the isolated microglia must be immediately plated. If the cells/media remain still (either in a T-75 flask or conical tube), the microglia are likely to reattach to the bottom of the container.

31. Centrifuge collected cells/media for 4 min 30 s at 180 rcf.
32. Aspirate media from the conical tube without disturbing the cell pellet and resuspend cells in 10% FBS/DMEM/PS media (warmed to 37°C).
33. Count the number of cells collected using a hemocytometer or other similar cell counting method.

***Note:*** The expected number of microglia obtained after shaking-off is approximately 3.5 × 10^5^ cells per cortex dissected from one mouse pup.

34. After counting cells, media should be added to the conical such that 1 mL of cell/media resuspension can be added to each well in a 12-well plate.
  a. It is suggested that the plated cell density is 5 × 10^4^ cells/well in a 12-well plate.

***Note:*** (Optional) Add additional 10% FBS/DMEM/PS media so there is 1 mL of media total per well.

35. After adding the 1 mL cell suspension to each well, tilt the 12-well plate so that the microglia are evenly distributed across the top of the coverslip in each well. Cells may also attach to the bottom of the wells, but the majority will attach to the top of the coverslips.

***Note:*** Note the density of the cells plated in each well. For the best results, plate no less than 3 × 10^4^ cells/well.

36. Incubate the cells for 24 h at 37°C prior to LPS treatment. After 24 h, if cells are not attached, please consult troubleshooting problem 2.

**CRITICAL:** Prior to beginning an experiment and treating the newly plated microglia, cells should be checked for health and attachment. Any discrepancies between wells should be noted and taken into consideration when proceeding with the experiment. Examples of plated unhealthy and healthy cells are visualized in Figure 4. If wells have unhealthy cells, please consult troubleshooting problem 1.

### LPS treatment to microglia

**Timing: 30 min hands-on, up to 25 h total**

Please refer below to Table 1 for LPS stock dilution and Table 2 for an example of LPS treatment preparation.

***Note:*** For LPS treatment and during the rest of the experiment, 1% FBS/DMEM/PS is used as a higher percentage of FBS can interfere with experimental conditions. Warm 1% FBS/DMEM/PS at 37°C before adding to wells.

***Note:*** As a control for LPS treatment, we recommend using the same volume of 1× dPBS.

37. Using the 1 mg/mL LPS Stock (see materials and equipment), prepare LPS treatments according to Tables 1 and 2.

***Note:*** Revise the components of Table 2 according to desired conditions for individual experimental set-up.

38. Aspirate the media from the 12-well plate, making sure not to disturb the seeded cells. Wash the seeded cells with 1 mL 1% FBS/DMEM/PS media (warmed to 37°C).
39. Immediately prior to treating seeded cells, aspirate the remaining media and add 800 μL of the LPS or dPBS treatment(s) prepared in media (based on Table 2).

**Figure 5:**
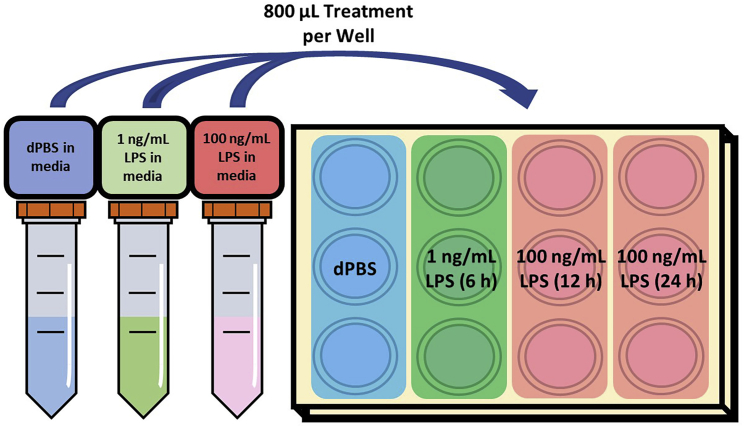
LPS treatment and timing strategy example for a single 12-well plate In this example, 800 μL of solution should be added to each well. This strategy can be revised according to the desired conditions for an individual investigator’s experimental set-up.

***Note:*** An example LPS treatment (dose and length of time) strategy is outlined in Figure 5.

40. Incubate treated cells at 37°C for the desired length of time (suggested: 6, 12 and/or 24 h, see Figure 5).

**Table 1:** LPS stock dilution example

LPS working stock concentration	Components
10 μg/mL	10 μL 1 mg/mL LPS Stock + 990 μL saline
100 ng/mL	10 μL 10 μg/mL LPS stock + 990 μL saline

**Table 2:** LPS treatment preparation example

Final LPS concentration	Components
100 ng/mL	40 μL 10 μg/mL LPS + 4 mL 1% FBS/DMEM/PS
1 ng/mL	40 μL 100 ng/mL LPS + 4 mL 1% FBS/DMEM/PS
0 ng/mL	40 μL dPBS + 4 mL 1% FBS/DMEM/PS

### Phagocytosis assay in microglia

**Timing: 30 min hands-on, 2 h 30 min total**

***Note:*** Before performing the phagocytosis assay, microglia seeded in a 12-well plate can be treated with LPS at the dosage and for the length of time (6, 12, and/or 24 h) of interest. An alternate form of microglia activation can be used such as IFNγ+TNFα.^9^ These pro-inflammatory stimulants result in microglia transitioning from basal state in which microglia surveil the environment to an activated pro-inflammatory state. This activated state will result in increased phagocytotic activity in microglia. If LPS stimulation fails to activate microglia, refer to troubleshooting problem 5.

41. Prepare 10 μL per well of bead treatment solution by mixing latex bead stock in a 1:10 ratio with 1% FBS/DMEM/PS.
  a. For example, for 12 wells, prepare 120 μL by adding 12 μL latex bead stock to 108 μL 1% FBS/DMEM/PS.
  b. This prepared volume accounts for extra treatment solution as only 8 μL of the solution well be added per well.
42. Add 8 μL of bead mixture directly to treatment media already in wells, tilting plate to distribute evenly within the well.

***Note:*** For appropriate treatment controls, incubate one well with 1% FBS/DMEM/PS prepared without fluorescent beads.

43. Incubate plates at 37°C for 2 h then aspirate beads and media without disturbing cells/cover glass.
44. Rinse wells with 1 mL 1× dPBS. Aspirate and repeat for two total washes with 1× dPBS.
45. Aspirate 1× dPBS from wells and add 1 mL 4% paraformaldehyde (PFA) (prepare 4% PFA as described above in materials and equipment).
46. Incubate wells with 4% PFA for 10 min at 37°C to fix cells.
47. Wash wells twice with 1 mL 1× dPBS.
48. Aspirate 1× dPBS from wells and add 1 mL 1× dPBS to each well for storage.
49. Protect plates from light by wrapping plates in aluminum foil and store at 4°C.
  a. Plates can be stored at 4°C for up to 1 month before visualization and analysis.

### Fluorescent bead treatment to microglia for phagocytosis

**Timing: Day 1 – 30 min hands-on, 2 h total(Step 50 - 55)**

**Timing: Day 2 – 45 min hands-on, 3 h 30 min total (**S**teps56**–**65)**

**Figure 6:**
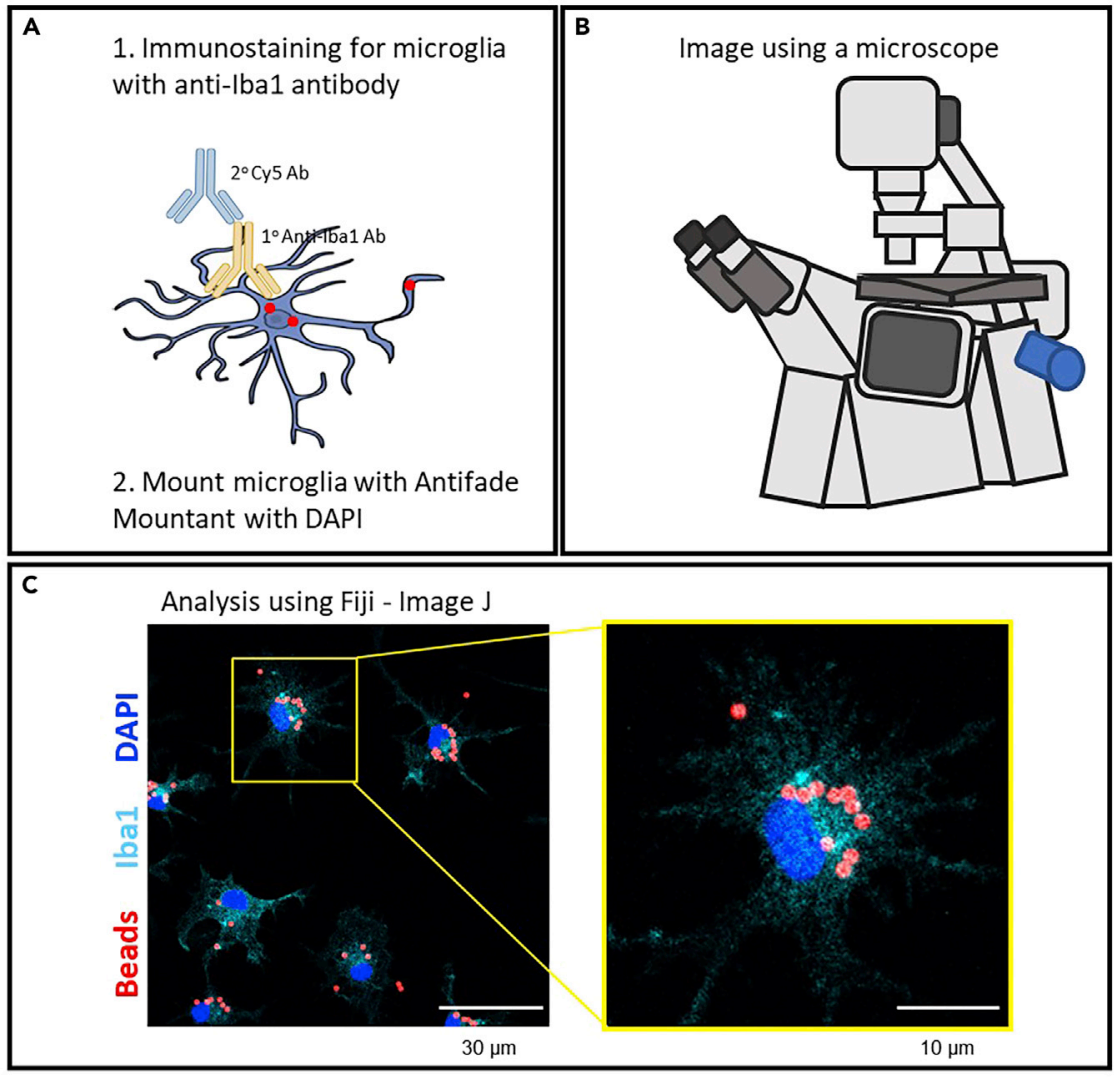
Visualization of microglia phagocytosis of fluorescent beads (A and B) Schematic depiction of immunostaining, mounting, and imaging steps. (C) Visualization of WT microglia phagocytosis of fluorescent beads after staining. Images are labeled with beads (red), microglia (Iba1, cyan), and nuclei (DAPI, blue). Higher magnifications of the left images (yellow box) are shown on the right panel. Scale bars for left image and right image represent 30 μm and 10 μm, respectively. Cells were imaged at 20× magnification and analyzed using Fiji – ImageJ software. Microglia images are adopted from a previous publication by Parrott et al. (2021).^1^

***Note:*** Iba1 is a microglia- and macrophage-specific protein marker commonly used when immunostaining microglia. However, Iba1 alone does not confirm the present of microglia and exclude a potential macrophage contamination. Therefore, other antibodies can be used in addition to anti-Iba1 to confirm microglia and should be selected based on the investigator’s need.

50. Aspirate 1× dPBS storage solution from wells without disturbing cells and the coveslip.
51. Incubate coverslips with blocking solution for 1 h 30 min at 18°C–26°C, covered to protect from light.
52. While incubating, prepare 70 μL of 1° ab solution per coverslip by adding Anti-Iba1, Rabbit (RB, 0.5 mg/mL, RRID: AB_839504) to blocking solution at a 1:500 ratio (Figure 6A).
  a. For example, for 12 wells, prepare 840 μL by adding 1.68 μL Anti-Iba1, Rabbit to 840 μL of blocking solution.
  b. This prepared volume accounts for extra 1° ab solution as only 60 μL of the solution will be added per coverslip.
  c. This 1° ab solution needs to be placed onto parafilm stretched over a 12-well plate in 60 μL “bubbles” for each coverslip.

Antibody for P2RY12, a receptor downregulated during inflammatory response, is specific to identify non-activated microglia^10^ although due to the LPS-induced activation of microglia suggested in this protocol, P2RY12 might not be an ideal target for identifying activated microglia without an additional primary antibody. Anti-transmembrane protein 119 (TMEM119) antibody is commonly used to distinguish microglia from peripheral macrophages, which would provide information on a potential macrophage contamination.^11^

53. Each coverslip needs to be removed from its well using forceps and placed cell-side down into “bubble” on the parafilm. Carefully place the lid of the 12-well plate over the coverslips.
54. With forceps, carefully pick up the coverslips and place each cell-side down into the “bubble” on the parafilm. Cover with 12-well plate lid.
55. Incubate in the 1° ab solution for 8–18 h at 4°C, covered to protect from light.

***Note:*** To differentiate background signal from the specific Anti-Iba1 signal, a negative control can be used. Prepare the negative control by adding Rabbit IgG Isotype control (5 mg/mL, RRID: AB_2532938) to blocking solution at a 1:5000 ratio then incubate the desired coverslips with the same method as described in steps 52–55.

56. Return coverslips to the wells of a 12-well plate.
57. Add 1 mL 1× PBS to wells to wash coverslips. Incubate for 5 min, covered, on a rocker. Aspirate 1× PBS and repeat for a total of 3 washes.
58. While washing, prepare 70 μL of 2° ab solution per coverslip/well by adding Cy™5 AffiniPure Donkey Anti-Rabbit IgG (H+L) (1.5 mg/mL, RRID: AB_2340607) to blocking solution at a 1:500 ratio.
  a. For example, for 12 wells, prepare 840 μL by adding 1.68 μL Donkey Anti-Rabbit IgG (H+L) to 840 μL of blocking solution.
  b. This prepared volume accounts for extra 2° ab solution as only 60 μL of the solution well be added per well.
  c. As with the blocking and 1° ab solution, the 2° ab solution needs to be placed onto parafilm stretched over a 12-well plate in 60 μL “bubbles” for each coverslip.
59. With forceps, carefully pick up the coverslips and place each cell-side down into the “bubble” on the parafilm. Cover with 12-well plate lid.
60. Incubate in the 2° ab solution for 1 h 30 min at 18°C–26°C, covered to protect from light.
61. Return coverslips to the wells of a 12-well plate. Wash coverslips with 1 mL 1× PBS per well and incubate for 5 min. Repeat for a total 3 washes.
62. Using forceps, remove each coverslip from the wells and mount on microscope slides with 1–2 drops of Invitrogen™ ProLong™ Gold Antifade Mountant with DAPI (DAPI visualizes nuclear DNA. DAPI staining is used as a nuclei marker that can identify cells).
  a. Mount 2–3 coverslips on each slide.
63. Allow slides to dry at 18°C–26°C for 1–2 h, covered.
64. Coat the edges of each coverslip on the slide with a thin layer of clear nail polish. This seals the cover glass to the slides.
65. Allow nail polish to dry at 18°C–26°C for 8–18 h, covered. Store slides at 4°C for long term storage.

***Note:*** Samples can be visualized on a Zeiss Confocal microscope or Zeiss fluorescence microscope with Apotome using Zeiss ZEN software (Figures 6B and 6C). Image analysis and phagocytosis quantification can be completed using Fiji - ImageJ software. See imaging and quantification below for additional information.

***Note:*** While fluorescent imaging is used in this protocol, an additional method for bead quantification in cells is flow cytometry, which provides the benefit of rapid analysis with a higher throughput. Our protocol uses immunostaining, fluorescent imaging, and manual counting to analyze the number of phagocytosed beads per cell and visualize the morphology of microglia.

### Imaging and quantification of phagocytosed fluorescent beads in microglia

**Timing: 2 h hands-on, 2 h total (depending on the sample size, it can be shorter or longer)**

66. Image microglia and beads using microscope; a Zeiss Confocal microscope or a Zeiss fluorescence microscope with Apotome and a 20× lens.
67. Prepare for imaging by determining an exposure time for each channel to be used; DAPI, fluorescent beads, and microglia.

***Note:*** This exposure time needs to remain constant between imaging different slides in the experiment.

68. Determine the Z-stack number and Z-stack interval that will be used for imaging.

***Note:*** Since microglia are typically about 10–15 μm thick and the diameter of the beads have a 0.5 μm mean particle size, we recommend the Z-stack interval to be between 0.5–1 μm to easily differentiate between beads. We recommend 10–15 Z-stacks depending on the chosen interval.

69. To begin imaging, find an area of the coverslip that includes multiple cells. Set up the first Z-position and last Z-position by focusing up and down using imaging software. Collect 2–3 Z-stack images per coverslip.

***Note:*** Once image parameters have been optimized, all cells need to be imaged using the optimized exposure time, Z-stack size and Z-stack interval so that cells in the same experiment can be compared.

***Note:*** For each genotype, treatment, and treatment length, 2–3 coverslips are plated, treated, assayed and immunostained for analysis. Following immunostaining, each coverslip is imaged and 2–3 images are collected for quantification per coverslip.

70. Following imaging, count beads per microglia using Fiji - ImageJ software.
  a. Within each image, assess every microglia (Iba1/DAPI positive) for co-localization of beads (i.e., 0+ beads) by viewing the images in each Z-stack.
  b. The Z-stack images provide information on bead size and shape so that individual beads can be identified within range of the microglia marker.
71. All beads per microglia values are averaged for each treatment and treatment length then statistically compared between each genotype.

***Note:*** If latex beads do not fluoresce, please see troubleshooting problem 3, and if immunostaining appears to be nonspecific, please see troubleshooting problem 4. Following analysis, if treatment does not increase phagocytosis, please see troubleshooting problem 5.

## Expected outcomes

The first part of this protocol describes how to dissect and culture glial cells from PND 2 murine cortical tissue. Murine brain dissection at PND 2 can be challenging due to the brain size, however, practice helps with skill development. We recommend performing dissection and culture trial runs with practice mice before performing an experiment. These steps must be mastered to ensure ideal microglia isolation and growth. When cultured properly, this dissection is a consistent and reliable method to obtain viable primary microglia from PND 0 to PND 10 mice.

The second part of this protocol describes how to induce and visualize microglia phagocytosis activity accomplished by microglia isolation, LPS treatment, incubation with fluorescent beads, and immunostaining. When microglia are activated with LPS, it is expected that there will be phagocytosis of the fluorescent beads, with multiple beads often found in one cell. A lack of increased phagocytic response to LPS treatment is addressed in troubleshooting problem 5. Using the experiment described here, *Fmr1* KO primary microglia, following treatment with both 1 ng/mL LPS and 100 ng/mL LPS, had higher phagocytosis activity than WT microglia treated similarly (Parrott et al.^1^).

## Limitations

This protocol has been successfully used with mice PND 0 to PND 10. Cultures collected at later ages take longer to populate to a useable experimental quantity *in vitro*. Adult primary microglia isolation has been previously demonstrated, however, isolation tends to be more difficult than postnatal primary microglia isolation.^6^

While there are many advantages using an *in vitro* approach to assess microglial phagocytic activity, there are limitations as well. An *in vitro* experiment such as this lacks the typical cell-to-cell interactions of microglia and other cells observed *in vivo*. Many pathways and cell interactions involved in phagocytosis are potentially disrupted by isolating microglia which may impact microglia activity. Additionally, while LPS is commonly used as a model for sepsis shock, LPS does not directly cross the blood brain barrier when administered peripherally, therefore; *in vitro* LPS treatment of microglia does not directly mirror typical *in vivo* inflammation.^12^ Instead of using LPS, pro-inflammatory cytokines such as IFNγ+TNFα can also be used as mentioned in troubleshooting problem 5. Additionally, fluorescent beads are not a physiological material, but can be used as an indicator of phagocytosis. With the limitations of *in vitro* experiments, it is necessary to limit extrapolations of results to *in vivo* microglia activity.^13^

## Troubleshooting

### Problem 1

Mixed cultures do not grow, or cells appear to be unhealthy cells after plating.

### Potential solution

The CO_2_ percentage levels in the incubator may not be at 5% as identified by the incubator’s internal monitors. Recalibrate the incubator as needed to achieve a consistent environment of 37°C, 100% humidity, and 5% CO_2_/95% air by using a Bacharach CO_2_ Gas Analyzer Kit (Grainger, Item # 6T151, UNSPSC #41113102, UNSPSC v841113102, Mfr. Model #10-5000). This gas analyzer will directly measure CO_2_ levels in the incubator to confirm what is displayed by the incubator’s internal monitors.

If the cell density is too low when initially cultured, cells may not grow by DIV 14. If the flask appears empty or less than 50% confluent by DIV 14, the cell culture preparation is not successful. To increase the number of cells isolated for the initial culture, either increase the number of pups dissected or use a smaller flask, such as a T-25 flask (25 cm^2^ culture area). If 2 pups at PND 2 do not provide enough cells for a T-75 flask, increase the number of pups while maintaining the 1:1 gender ratio. At later PND ages, 2 pups should be sufficient for culturing in a T-75 flask.

If the mixed glial culture is failing to grow when using pups older than PND 2–3, the media used can be changed for culturing and plating. Instead of using 10% FBS/DMEM/PS media and 1% FBS/DMEM/PS media (described above in materials and equipment), use 15% FBS/DMEM/F-12/PS media and 1% FBS/DMEM/F-12/PS. DMEM/F-12 media (ThermoFisher Scientific, Cat. No. 11320082) contains Ham’s F-12 medium, a nutrient mixture that provides additional supplements to the cells. Additionally, increasing the FBS concentration provides a higher supplement concentration that better supports the metabolic requirements of glial cells.

The culture seeding density may be too low. To a 12-well plate, we suggest the optimal cell density for plating for a phagocytosis assay should be about 5 × 10^4^ cell/well. This density is optimal for analysis purposes as well as cell viability. In our experience, the minimum viable cell density for plating is about 3 × 10^4^ cell/well. However, if the cells are consistently failing to grow or are unhealthy when plating an optimal density of cells, consider allowing more DIV for cells to reach 70%–80% confluence before seeding.

Cells may be unhealthy when adding to the 12-well plate. Before shaking, cells should be examined underneath a microscope to ensure their health and density. An example of healthy cells ready for shaking are demonstrated in Figure 3 from DIV 10–14. Alternatively, cells may not be viable after shaking if shaken for longer than 3.5 h or if the temperature is too low. After shaking, it is expected that the cell density of microglia from 4 cultured brains resuspended in 2–3 mL of media will be a least 16 × 10^4^ cells/mL. If this number is much lower, cells density may be too low for plating. In that case, the resuspended cells should not be used for the phagocytosis assay. A new culture must be made, or the T-75 flask should be given 1 week for microglia proliferate and re-shaken. After cells have been plated, cells should be examined underneath a microscope to ensure their health and density. Examples of healthy and unhealthy plated cells are demonstrated in Figure 4.

### Problem 2

Microglia do not attach to the coverslip.

### Potential solution

The 1× PLL coating may not have incubated for long enough or had not fully coated the cover glass. It is important to agitate the cover glass coated with 1× PLL about once per hour to ensure the entire coverslip is submerged. If the coverslip is not completely submerged using 600 μL 1× PLL, add more 1× PLL (up to 1 mL/well total) to submerge the coverslip.

### Problem 3

Latex beads do not fluorescence when imaged.

### Potential solution

Proper storage for the latex beads is necessary to preserve the fluorescent signal. The latex bead solution should be stored in a dry, well-ventilated area and protected from light at all times. Additionally, the beads used in the experiment may be changed from fluorescent red to an alternate fluorescent color to suit the needs of the investigator.

### Problem 4

Nonspecific immunofluorescent staining.

### Potential solution

Proper storage is necessary to maintain the reactivity of the antibody. Contact the antibody supplier to optimize storage conditions if needed. Depending on the needs of the investigator, the antibody targets may be adjusted and optimized as needed. However, these targets confirm cells (nuclei staining: DAPI) and identify cell type (microglia: Iba1).

Antibody incubation durations and concentrations used for immunostaining may need to be optimized when establishing this protocol within a new lab. The conditions and antibody concentrations used in this experiment can be optimized within the lab for the use of the specific antibodies. Additionally, the temperature at which the antibody is incubated can be adjusted. For example, instead of incubating the 1° antibody solution at 4°C for 8–18 h, try incubating it at 18°C–26°C for 4–6 h.

### Problem 5

Microglia do not have increased phagocytosis activity in response to LPS treatment.

### Potential solution

This problem is most likely due to low phagocytosis activity in microglia, due to the age and/or health of the cells. Previous work demonstrated that it is possible to successfully harvest microglia following repetitive shake-offs from a single culture, however, the magnitude of responsiveness to LPS treatment decreases with each shake-off.^3^ To minimize this impact on experiments, only compared data collected from the same experiment and same shake-off. Additionally, try to use first or second shake-offs and limit the use of third and fourth shake-offs when possible.

Alternate forms of cell activation may be used to assess phagocytic activity. Other pro-inflammatory stimuli such as IFNγ or TNFα cytokines have also been used to stimulate microglia cultured from rats, however, the cytokines were found to be less effective at creating a robust-inflammatory response in microglia.^9^

## Resource availability

### Lead contact

Further information and requests for resources and reagents should be directed to and will be fulfilled by the lead contact, Hye Young Lee (leeh6@uthscsa.edu).

### Materials availability

The study did not generate new unique reagents.

## Data Availability

All data reported in this paper will be shared by the lead contact upon request. This paper does not report original code. Any additional information required to reanalyze the data reported in this paper is available from the lead contact upon request.
